# P2RY13 is a prognostic biomarker and associated with immune infiltrates in renal clear cell carcinoma: A comprehensive bioinformatic study

**DOI:** 10.1002/hsr2.1646

**Published:** 2023-12-01

**Authors:** Jie Chu, Wei Liu, Xinyue Hu, Huiling Zhang, Jiudong Jiang

**Affiliations:** ^1^ Department of Oncology The First People's Hospital of Ziyang Ziyang China; ^2^ Department of General Family Medicine The First People's Hospital of NeiJiang NeiJiang China; ^3^ Department of Clinical Laboratory, Kunming First People's Hospital Kunming Medical University Kunming China; ^4^ Department of Surgery The First People's Hospital of ZiYang Ziyang China

**Keywords:** biomarker, clear cell renal cell carcinoma, GEO, P2RY13, TCGA

## Abstract

**Background and Aims:**

Clear cell renal cell carcinoma (ccRCC) is a common and aggressive form of cancer with a high incidence globally. This study aimed to investigate the role of P2RY13 in the progression of ccRCC and elucidate its mechanism of action.

**Methods:**

Gene Expression Omnibus and The Cancer Genome Atlas databases were used to extract gene expression profiles of ccRCC. These profiles were annotated and visualized by Gene Ontology (GO) and Kyoto Encyclopedia of Genes and Genomes (KEGG) functional enrichment analyses, as well as Gene Set Enrichment Analysis (GSEA). The STRING database was used to establish a protein–protein interaction network and to analyze the functional similarity. The GEPIA2 database was used to predict survival associated with hub genes. Meanwhile, the TIMER2.0 database was used to assess immune cell infiltration and its link with the hub genes. Immunohistochemistry (IHC) was used to determine the difference between ccRCC and adjacent normal tissue.

**Results:**

We identified 272 differentially expressed genes (DEGs). GO and KEGG analyses suggested that DEGs were primarily involved in lymphocyte activation, inflammatory response, immunological effector mechanism pathways. By cytohubba, the 20 highest‐scoring hub genes were screened to identify critical genes in the protein–protein interaction network linked with ccRCC. Resting dendritic cells, CD8 T cells, and activated mast cells all showed a significant positive correlation with these hub genes. Moreover, a higher immune score was associated with increased prognostic risk scores, which in turn correlated with a poorer prognosis. IHC revealed that P2RY13 was expressed at higher levels in ccRCC compared to para‐cancer tissues.

**Conclusion:**

Identifying the DEGs will aid in the understanding of the causes and molecular mechanisms involved in ccRCC. P2RY13 may play a pivotal role in the progression and prognosis of ccRCC, potentially driving carcinogenesis though immune system mechanisms.

## 1 INTRODUCTION

Clear cell renal cell carcinoma (ccRCC) is the most prevalent subtype of renal cell carcinoma,^1^ accounting for the majority (75%) of all kidney cancer cases,^2^ and its prevalence is increasing.^3^ Owing to postoperative metastases and medication resistance,^4^ ccRCC has one of the highest mortality rates among all malignancies,^5^ and its incidence and mortality have grown fast over the last 20 years.^6^ It also has an extremely poor prognosis, with a 5‐year survival rate of approximately 96% for early‐stage ccRCC and <10% for late‐stage ccRCC.^7^ Addressing the morbidity and mortality of this cancer remains a significant clinical hurdle. Thus, comprehending the process of ccRCC development and developing reliable predictors linked with tumor stage and prognosis are critical for appropriate diagnosis and therapy of the disease.

Many markers have been reported for ccRCC. For example, ANGPTL4 has been demonstrated to be a diagnostic marker for both primary and metastatic ccRCC.^8^ CA IX may represent a putative marker related to ccRCC,^9^ as s100A1 was reported to be differentially expressed in ccRCC and papillary ccRCC.^10^ In addition, several genes have been linked to the development of ccRCC, including *VHL*, a ubiquitin ligase, and tumor suppressor gene, as well as *c‐Met* and *ltd*,^11^ which is downregulated in ccRCC and is an early diagnostic marker.^12^ P2RY13 a G protein‐coupled receptor that reacts to extracellular purine and pyrimidine nucleotides, negatively regulates adenylate cyclase activity.^13^ Encoded by 354 amino acids (aa), it is linked to the development of inflammation and immune conditions.^14^ While P2RY13 is particularly sensitive to ADP, it can be triggered by both ADP and ATP. Research indicates a crucial function of P2RY13 in inflammation and immune imbalance.^15^ Several studies have reported that P2RY13 is a key regulator of cholesterol transport and hepatic HDL endocytosis and is involved in bone formation, remodeling, cell survival, and neuroprotection.^13^ Animal experiments have shown that P2RY13 protected the host from viral infection, highlighting its potential involvement in immune responses. Our preliminary bioinformatic preliminary showed elevated P2Y13 expression in ccRCC patients. Given the ambiguous role of P2RY13 in ccRCC, we delved into its mechanism in ccRCC and examined its correlation with prognosis, focusing on the messenger RNA (mRNA) expression value of P2RY13.

The significance of the tumor microenvironment in ccRCC has been previously studied. The survival outcomes of patients with ccRCC were investigated using quantitative polymerase chain reaction (qPCR) to determine the underlying gene expression profile^16^ in 537 individuals with ccRCC. In that specific study, qPCR methods identified only two tumor phenotypes: proliferative and invasive. This highlights the need for high‐throughput techniques to fully evaluate all tumor phenotypes and comprehensive immune profiles. Several recent studies, including those employing microarray analysis, have pointed to differentially expressed genes (DEGs) and functional pathways as key players in ccRCC development.^17^ Inconsistent results in these studies could be due to different microarray technologies and sample numbers. To address these inconsistencies, more research is needed to identify more powerful and reliable diagnostic biomarkers. Owing to the lack of external validation in the training set, a larger cohort of patients with ccRCC is needed to confirm the findings.

In the present study, to assess the potential prognostic value of P2RY13 expression in human ccRCC, we analyzed gene expression profiles from The Cancer Genome Atlas (TCGA) and the Gene Expression Omnibus (GEO) databases, using the Gene Ontology (GO) enrichment and Gene Set Enrichment Analysis (GSEA) pathways to analyze the biological processes (BPs) involved in protein–protein interaction (PPI) networks to evaluate potential biological interaction networks, which may provide insights into the molecular mechanisms of ccRCC.

## 2 MATERIALS AND METHODS

### 2.1 Data download and data pre‐processing

The primary RNA sequencing (RNA‐seq) data and corresponding clinical information of ccRCC patients were downloaded from the TCGA cancer genomics program (https://portal. https://gdc.cancer.gov/repository). The GDC Hub data from online USUC Xena database (http://xena.ucsc.edu/) based on TCGA cohorts were used to analyze the RNA‐seq data in ccRCC. The GEOquery package^18^ of R software (version 3.6.5, http://r-project.org/) was used to download samples from the GEO (https://www.ncbi.nlm.nih.gov/geo/) database with a reliable source of ccRCC expression profiling data set GSE53757,^19^ and the samples in the data set were from *Homo sapiens* and platform based on GPL570 [HG‐U133_Plus_2] Affymetrix Human Genome U133 Plus 2.0 Array. While the original data set is TCGA‐KIRC, the curated subset from XENA used in our research is termed TCGA‐ccRCC. TCGA contained UCSC Xena ccRCC FPKM data and related clinical information, and GSE53757 data set included 72 ccRCC patient samples and 72 normal samples, while TCGA included 522 ccRCC patient samples and 71 normal samples in the data set. Normal samples were included in this study (Supporting Information S1: Table S1). The raw data of the GSE53757 data set were read using the affy package,^20^ background‐corrected by RMA, and normalized by data to obtain the gene expression matrix of this data set. TCGA correlation data were processed with R. HGNC,^21^ known as the HUGO Gene Nomenclature Committee, is responsible for providing unique, standardized, and widely disseminated symbols for all genes on the human genome, including protein‐coding genes, lncRNA genes, methyl genes, and other genes; for each human gene, there is a numerical identifier in the HGNC mRNA and microRNA expression profiles that can be obtained using the mRNA and microRNA gene annotation files of HGNC.

### 2.2 Expression characteristics and survival analysis of P2RY13 gene

The mechanism of P2RY13 in ccRCC remains unclear; therefore, we investigated the mechanism of its action in ccRCC and its relationship with prognosis using the mRNA expression value of P2RY13. The GEPIA2 “Survival” module^22^ was first used to perform differential expression analysis and prognostic analysis of P2RY13 gene pair in ccRCC data set in TCGA by using default settings, and then the TIMER2.0^23^ database was then used to show the distribution of P2RY13 expression in the whole body as default settings.

### 2.3 Identification of DEGs

We divided the GSE53757 and TCGA ccRCC data sets into high‐ and low‐expression groups using quartiles of expression values of P2RY13 and performed differential expression analysis with the lowest expressed quartile and the highest expressed quartile groups.^24^ The GSE53757 and TCGA data sets were screened for DEGs using the limma package using default settings,^25^ and the volcano plot of DEGs was plotted using ggplot2 (Ito and Murphy, 2013) to demonstrate the differential expression of DEGs. DEGs satisfied adj.*p* < 0.05 and |log_2_FC|> 1.5. We used the intersection of TCGA and GSE53757 DEGs for the next analysis and plotted the intersection of the two data sets using the R package Venn Diagram.^26^

### 2.4 GO and KEGG analysis

GO is a database established by the Gene Ontology Consortium (2019) over 20 years ago to create a semantic vocabulary standard for qualifying and describing gene and protein functions applicable to various species and updated as research progresses. It is divided into three major categories: molecular function (MF), biological process (BP), and cellular components (CC). KEGG is a comprehensive database that integrates genomic, chemical, and systemic functional information^27^ and has a database called KEGG Pathway, which specifically stores information about gene pathways in different species. Metascape^28^ is a web tool that provides a variety of functions, such as gene enrichment analysis and protein interaction network analysis. The website integrates more than 40 gene function annotation databases and provides diverse visualizations. We used Metascape to perform GO/KEGG functional enrichment analysis of DEGs, selecting functions with *p* < 0.01, minimum count of 3, and enrichment factor >1.5. We also used the R package Pathview^29^ to visualize the more important pathways in KEGG and used the R package ggplot2 to visualize the more important functions in GO.

### 2.5 GSEA analysis

GSEA^30^ or gene set enrichment analysis is based on the idea of using predefined gene sets (usually from functional annotations or results of previous experiments) to rank genes according to their differential expression in two types of samples and then testing whether a predefined set of genes is at the top or bottom of the ranking table enrichment. We used the clusterProfiler package^31^ to perform gene set enrichment analysis of the gene expression matrix using the GSEA method on the gene expression profiles of TCGA, selecting “c2.cp.kegg.v7.4.symbols.gmt” and “c5.go.bp.v7.4.symbols.gmt” as the reference gene sets^32^ and a significance level of *p* < 0.05.

### 2.6 Construction of PPI network for protein analysis and identification of hub genes

The STRING^33^ database can search for interactions between known and predicted proteins. The parameters of STRING were set as below: all seven active interaction sources, medium confidence, and query proteins only. We used the DEGs obtained from differential expression analysis and placed them into the STRING database to obtain their protein interaction networks and then used the networks to identify genes that interacted more strongly with other genes and visualize them using Cytoscape^34^ software. Using the MCODE^35^ plugin to identify its sub‐networks and based on the score, we obtained three sub‐networks with scores greater than 10, which we assumed might serve a specific function. Adapted to the hub gene with the cytohubba^36^ plugin, a total of 20 hub genes were obtained. TargetScan^37^ is a microRNA (miRNA) target gene prediction website, including miRNA target gene results for five species: human, mouse, Drosophila, nematode, and zebrafish. We obtained a total of nine significant microRNAs (the number of samples with expression value of 0 was less than 10), identified the target genes of the nine microRNAs using the TargetScan database, performed correlation analysis between microRNAs and DEGs, removed the genes with a Pearson correlation coefficient greater than 0 (*p* < 0.05), and used the intersection with the target genes to finally obtain the miRNA‐mRNA regulatory network and visualize with Cytoscape.

### 2.7 The relationship between immune cell infiltration and diagnostic markers

CIBERSORTx website (https://cibersort.stanford.edu/)^38^ is based on the principle of linear support vector regression to deconvolute the transcriptome expression matrix to estimate the composition and abundance of immune cells in a mixture of cells. We downloaded the original code and the corresponding immune cell files from the official CIBERSORT website and derived the immune cell infiltration matrix in R based on the gene expression profile of TCGA ccRCC and the immune cell files. We used the corrplot package to plot correlation heat maps to visualize the correlation of 22 immune cell infiltrations, the ggplot2 package to construct box line plots for visualizing the differences of the 22 immune cell infiltrations, and the igraph package^39^ to prepare correlation network plots of the immune cell infiltrations to visualize the interactions of the 22 immune cell infiltrations using *p* < 0.05 and |correlation coefficient｜>0.2 (correlation coefficient: correlation coefficient) as the interactions criteria. We correlated the obtained diagnostic markers with immune cell infiltration and visualized the results using the pheatmap^40^ package, drawing a heat map.

### 2.8 Identification of prognosis‐related genes from hub genes for corresponding survival analysis

One‐way Cox proportional risk regression analysis was performed on the hub genes obtained from PPI network analysis, and genes with *p* < 0.05 were selected. A multi‐factor Cox proportional risk regression analysis was performed for the six obtained genes to construct a prognostic risk assessment model. The prognostic risk of each sample in TCGA was scored according to the above model, and the cutoff value was the median value of the prognostic risk scores of patients in the training set. Patients were divided into different risk groups according to the cutoff value delineation, and survival analysis was performed, and the results were visualized based on the high‐ and low‐risk groups using the R survival^41^ package and the Survminer^42^ package. Furthermore, the prognostic risk scores of TCGA patients were analyzed by one‐way Cox proportional risk regression analysis and multi‐factor Cox proportional risk regression analysis with the clinical characteristics of the patients. The results were visualized with the R package forestplot^43^ to stratify the characteristics associated with the prognosis of ccRCC based on the prognostic risk score.

### 2.9 Diagnostic analysis of prognostic risk assessment model and prognostic genes

Patients were ranked from lowest to highest based on the scores derived from the prognostic risk assessment model and the tendency of the expression change of the six genes with the scores was observed using the heat map and score distribution curve after ranking in the GSE53757 and TCGA ccRCC data sets, respectively. The diagnostic values of the six genes were evaluated by clustering heat map analysis of the six prognostic genes. The R package ESTIMATE^44^ was used for tumor purity prediction, using expression profile data to predict stromal and immune cell scores and thus the content of these two types of cells. Finally, the tumor purity inside each tumor sample could be calculated. We used the ESTIMATE package to obtain the immune scores of patients and used a boxplot to show the relationship between prognostic risk scores in TCGA ccRCC and GSE53757.

### 2.10 Immunohistochemistry (IHC)

Kidney tissues were obtained following approved institutional ethical guidelines, paraformaldehyde‐fixed embedded sections, placed in automatic stainer for dewaxing; automatic immunohistochemistry pretreatment system instrument to complete antigen repair, PBST buffer rinse, dropwise addition of primary antibody P2RY13 (Catalog Number: 20335‐1‐AP, Proteintech), 4° refrigerator overnight; the next day, room temperature rewarming 30 min after PBST rinse; incubate dropwise with secondary antibody and rinse three times with PBST for 1 min/time. Diluted DAB was added dropwise to the slices and the intensity of colour development was observed. Hastings hematoxylin (SIGMA) was added dropwise for 1 min, then submerged in 0.25% hydrochloric alcohol (400 mL 70% alcohol + 1 mL concentrated hydrochloric acid) for 2 s, rinsed with tap water for 2 min, allowed to dry at room temperature and then the slides were sealed.

### 2.11 Statistical analysis

Statistical analysis was performed using SPSS 25.0 statistical software. Quantitative data were expressed as mean ± standard deviation (*x* ± *s*), using *t*‐test; qualitative data were expressed as frequency and percentage (%), using *χ* ^2^ test and Fisher's exact test. To evaluate the diagnostic accuracy of P2RY13, the ROC curve analysis based on sensitivity and specificity was conducted using the “pROC” package. The area under the curve (AUC) ranges from 1.0 (perfect diagnostic) to 0.5 (no diagnostic value).

## 3 RESULTS

### 3.1 Data download and preprocessing

First, the gene expression matrix of GSE53757 (Related information of data set was in Supporting Information S1: Table S1) was downloaded from the GEO official website using affy package, normalized, and processed based on RMA method; then the ccRCC patient data set (Related information of data set was in Supporting Information S1: Table S1) was downloaded from TCGA and preprocessed with R. Protein gene annotation files were downloaded from HGNC, and after matching, a total of 16,930 mRNAs were obtained in GEO and 19,070 mRNAs in TCGA.

### 3.2 Expression characteristics and survival effect of P2RY13 gene

**Figure 1:**
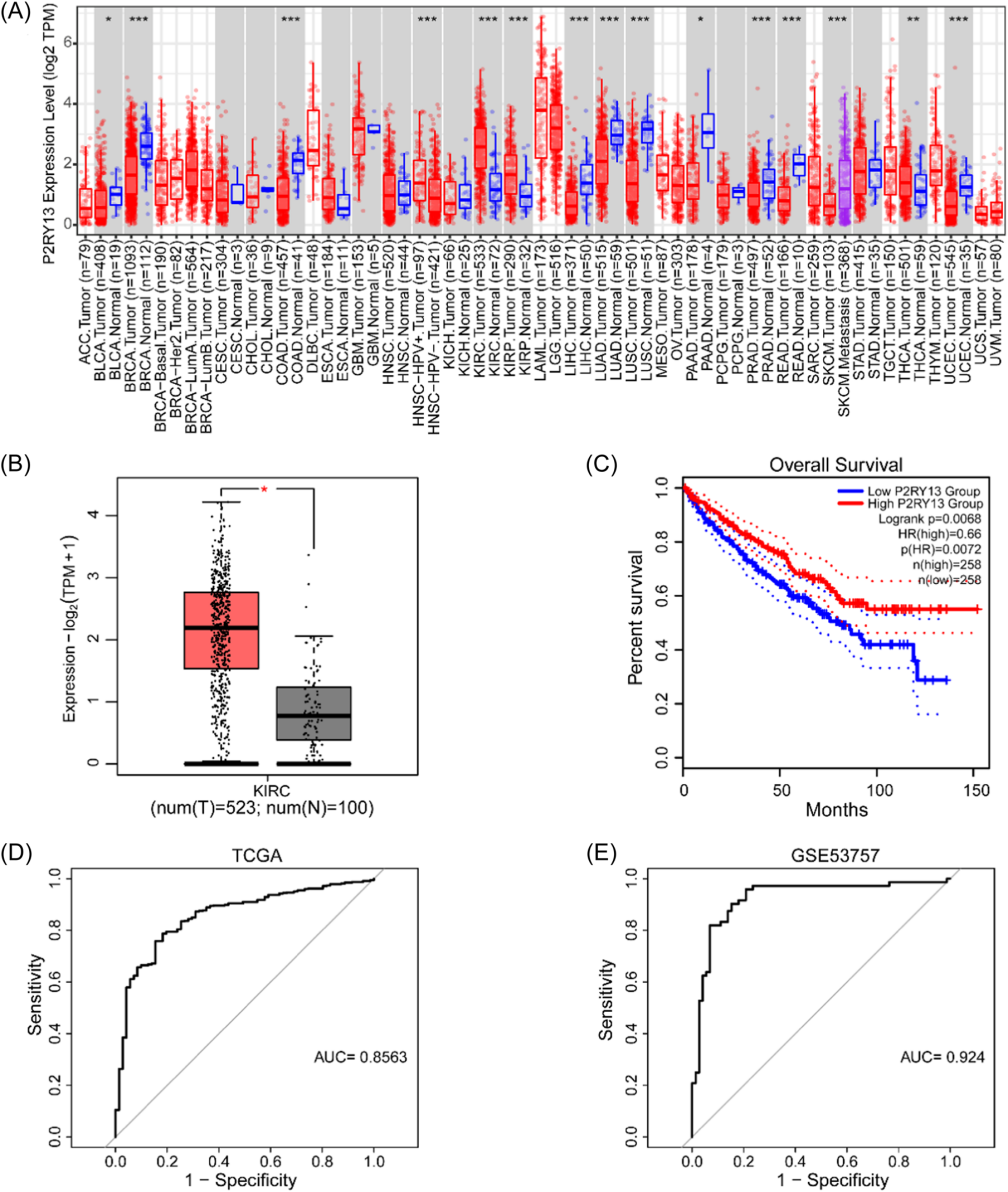
Differential expression of P2RY13. (A) The expression distribution map of P2RY13 gene between tumor and normal samples; (B) Box plot of P2RY13 gene in ccRCC; (C) gene survival analysis in ccRCC of P2RY13; (D, E) ROC curve of P2RY13. *p < 0.05, **p < 0.01, ***p < 0.001. ns, not statistically significant; num(N), normal sample number; num(T), tumor sample number.

First, we used the TIMER2.0 database for P2RY13 to show the expression distribution of P2RY13 in 33 cancers (Figure 1A). In cancers where adequate normal tissue data was available, GPC2 expression differed significantly in 15 cancer types compared to normal tissue. We then explored the differential expression and prognosis of this gene in ccRCC using GEPIA2 and found that the cancer group had higher expression than the normal group, containing TCGA normal and GTEx data (523 cancer samples and 100 normal samples) (Figure 1B), while the higher expression corresponded to a worse prognosis (Figure 1C). We then performed diagnostic analysis in GSE53757 and TCGA ccRCC based on the expression value of this gene (Figure 1D,E). It can be found that the Area Under Curve (AUC) values were greater than 0.8 in both sets, which further proves that the gene has a good diagnostic effect.

### 3.3 Differential expression analysis

**Figure 2:**
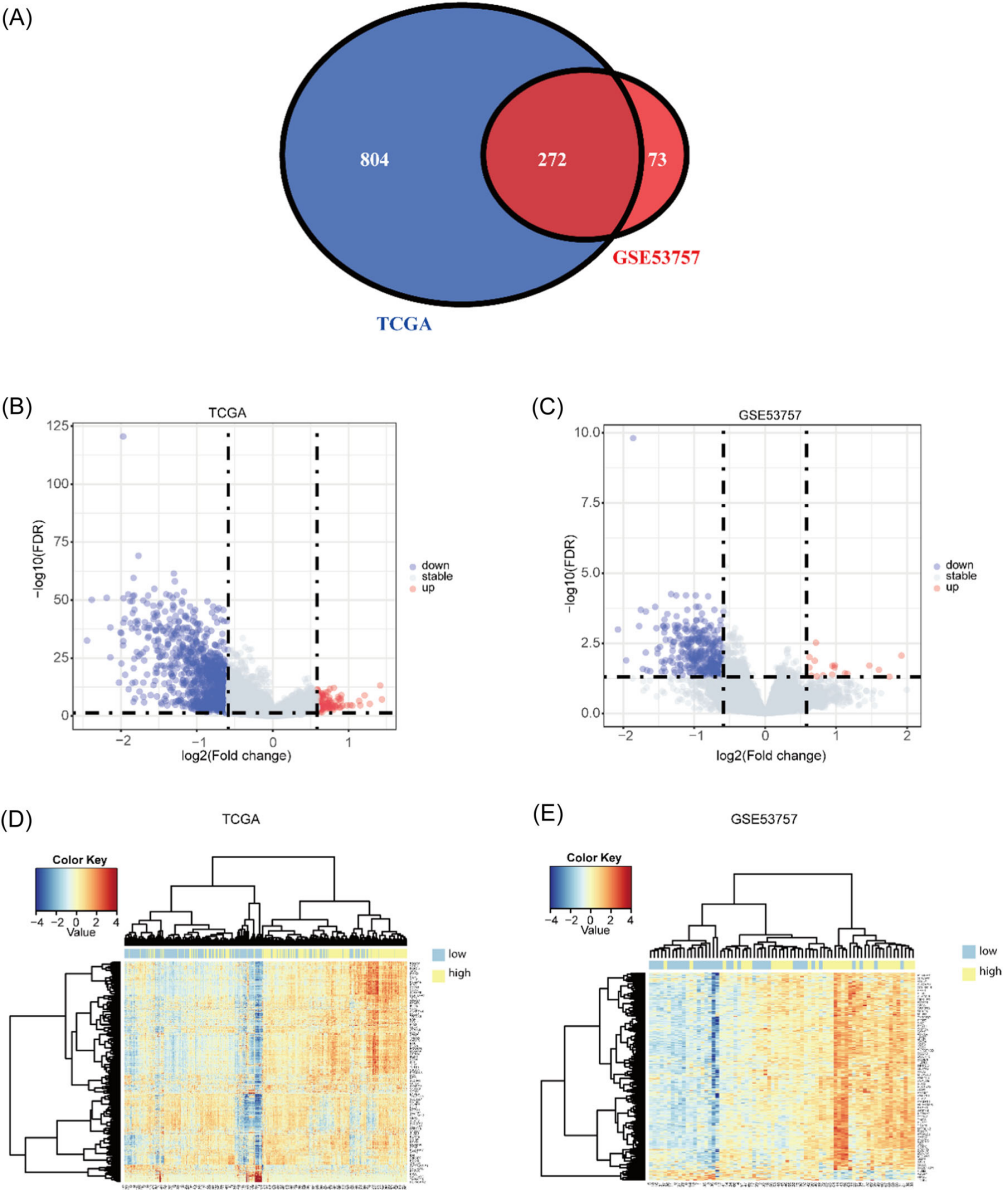
Differentially expressed genes (DEGs) analysis. (A) Venn diagram of DEGs from 2 data sets; (B) volcano plot of The Cancer Genome Atlas (TCGA) DEGs, red represents upregulated differential genes, blue represents downregulated differential genes, and grey represents no differential genes; (C) volcano plot of GSE57357 DEGs; (D) TCGA clustering heatmap; (E) GSE53757 clustering heatmap. Yellow represents the P2RY13 high‐expression group, and blue represents the P2RY13 low‐expression group.

In our study, our approach was to identify DEGs separately in each data set and then find the intersection of these DEGs. After data preprocessing, we divided the data set into high‐ and low‐expression groups according to the quartiles of expression values of P2RY13 and performed differential expression analysis with the lowest and highest expression quartiles. We used R package limma for differential expression analysis of the expression matrix, with |log_2_FC|>log21.5 and adj.*p* < 0.05 as the threshold screening, and a total of 345 DEGs, 17 upregulated genes, and 328 downregulated genes were extracted from the gene expression matrix in GSE53757. The distribution of the DEGs is shown as a volcano plot (Figure 2B). A total of 1076 DEGs were extracted from the gene expression matrix in TCGA, with 95 upregulated genes and 981 downregulated genes, and the distribution of DEGs is shown as a volcano plot (Figure 2C). We obtained 272 DEGs by taking the intersection of the difference sets of the two sets, which are displayed as a Venn diagram (Figure 2A). To provide a comprehensive visualization of the expression patterns of all 272 DEGs in relation to one another, we then performed a hierarchical clustering analysis of the 272 DEGs in GSE57357 and TCGA and found that the vast majority of P2RY13 low‐expression samples were clustered into one class, and P2RY13 high‐expression samples were clustered into one class (Figure 2D,E).

### 3.4 GO and KEGG analysis

**Figure 3:**
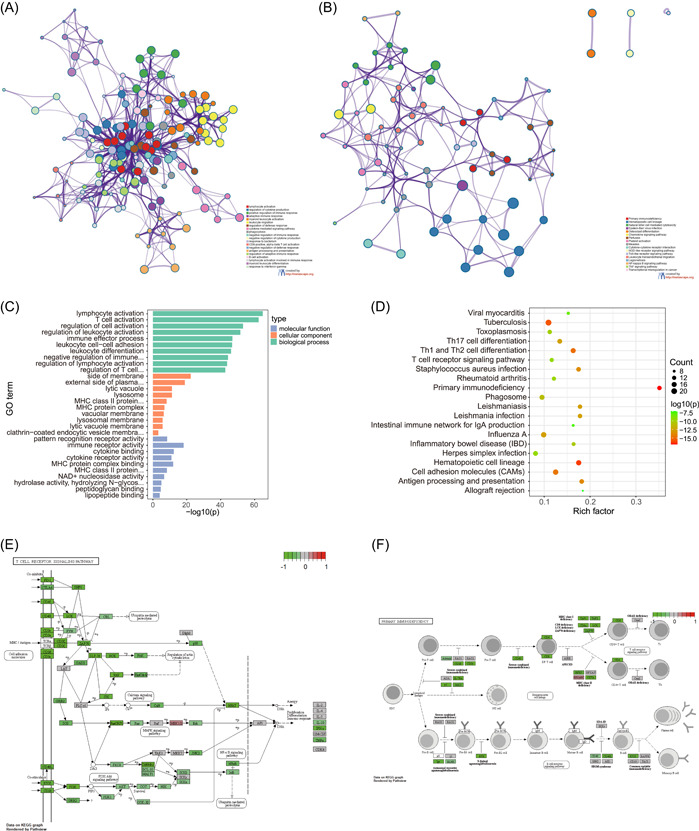
Functional enrichment analysis of differentially expressed genes (DEGs). (A) Network of top 20 Gene Ontology (GO) enrichment functions, the color represents cluster IDs, node represents an enriched term; (B) network of top 20 KEGG enrichment results, the color represents cluster IDs; (C) bar plot of GO enrichment functions, *p* value are shown by length of functional columns; (D) dot plot of top 20 KEGG enrichment results; (E) pathway map of T‐cell receptor signaling pathway; (F) pathway map of primary immunodeficiency.

We first performed a functional enrichment analysis of DEGs using Metascape to screen for function at *p* < 0.01, minimum count of 3, and enrichment factor >1.5. The results showed that DEGs were mainly associated with lymphocyte activation, inflammatory response, immune effector processes, pattern recognition receptor activity, immune receptor activity, membrane side, plasma membrane outer side, primary immunodeficiency, T‐cell receptor signaling pathway, and hematopoietic cell lineage (Figure 3) (Supporting Information S1: Tables S2 and S3). Detailed enrichment results are shown in Supporting Information S1: Appendix 1.

### 3.5 GSEA analysis

**Figure 4:**
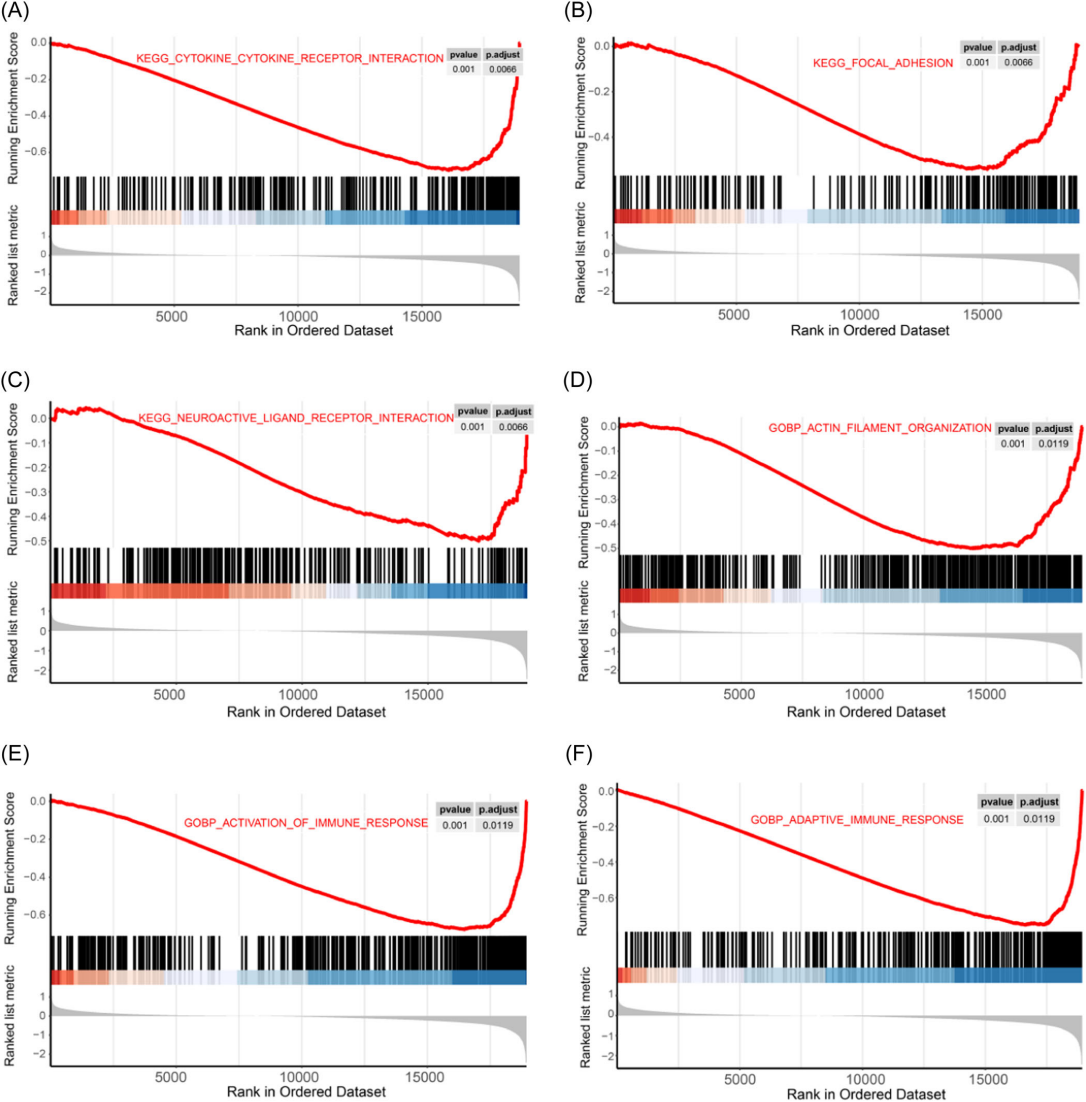
Gene Set Enrichment Analysis (GSEA) functional enrichment analysis. (A–C) Top three GSEA enrichment results for Kyoto Encyclopedia of Genes and Genomes (KEGG). (D–F) Top three GSEA enrichment results for Gene Ontology (GO). (A) KEGG cytokine receptor interaction; (B) KEGG focal adhesion; (C) KEGG neuroactive ligand–receptor interaction; (D) GOBP actin filament organization homeostasis; (E) GO biological process (GOBP) activation of immune response; (F) GOBP adaptive immune response.

We first downloaded the gene sets “c2.cp.kegg.v7.4.symbols.gmt” and “c5.go.bp.v7.4.symbols.gmt” from the GSEA website, and the GSEA function was used in the clusterProfiler package to perform GSEA functional enrichment analysis of the GSE38713 expression profile using “c2.cp.kegg.v7.4.symbols.gmt” and “c5.go.bp.v7.4.symbols.gmt” as reference gene sets (these two are more commonly used in functional enrichment). We used a *p* < 0.05 as the threshold to screen for differential functions, and the results of KEGG and GO enrichment are shown in Figure 4 and Supporting Information S1: Table S4, and the mainly enriched pathways included GO biological process (GOBP) actin filament organization, GOBP activation of immune response, GOBP adaptive immune response, KEGG cytokine receptor interaction, KEGG focal adhesion, and KEGG neuroactive ligand–receptor interaction. The detailed enrichment results are shown in Supporting Information S1: Appendix 2.

### 3.6 Construction of PPI network and miRNA‐mRNA network

**Figure 5:**
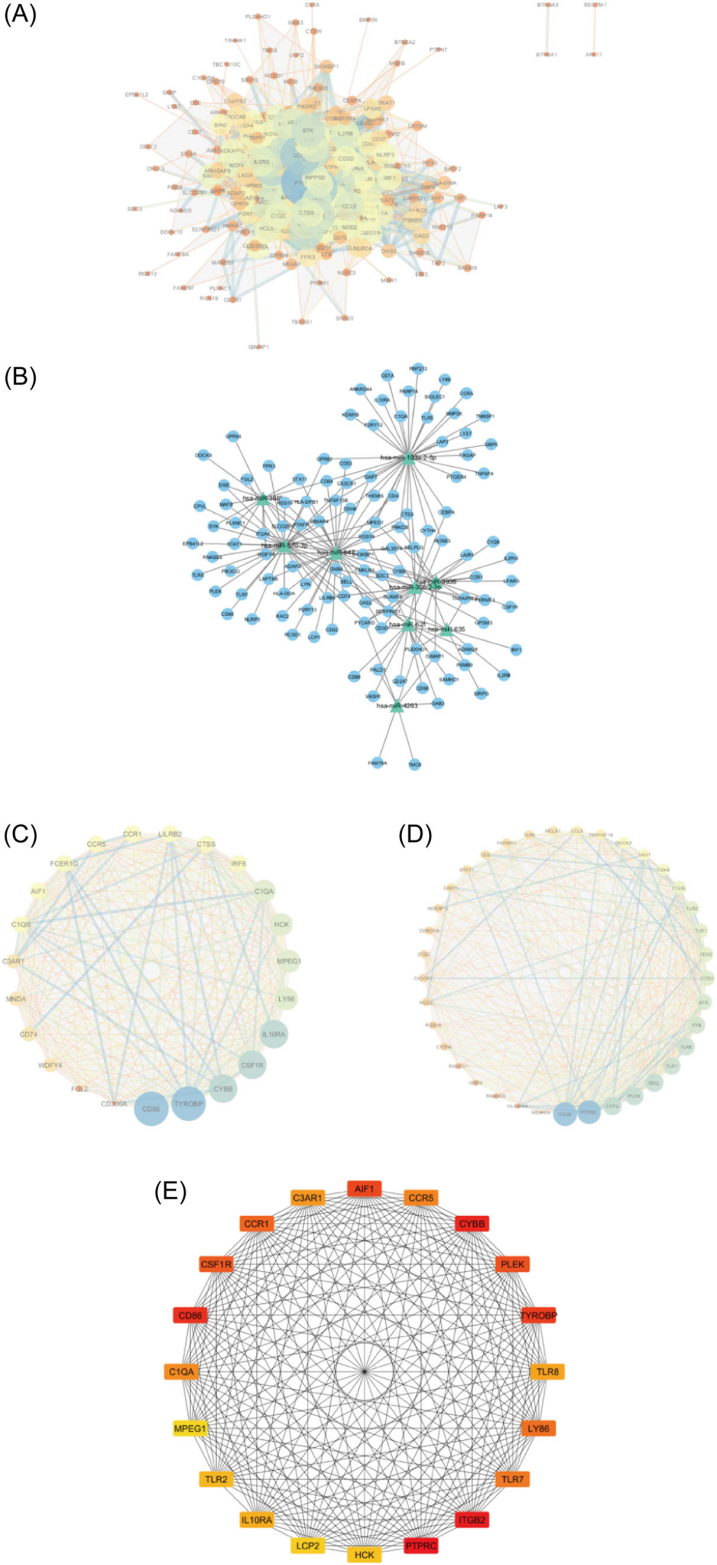
Protein–protein interaction (PPI) network. (A) PPI network obtained from STRING database; (B) microRNA‐messenger RNA (miRNA‐mRNA) regulatory network; (C, D) the two highest scoring subnetworks identified with the MCODE plugin, which are considered to be the two most important functional modules; (E) the 20 highest scoring hub genes obtained with the cytohubba plugin.

We placed the obtained DEGs into the STRING database to obtain their PPI networks and then used Cytoscape to identify and visualize the important genes that had strong interactions with other genes from the PPI networks (Figure 5A). Using the MCODE plug‐in, we identified the two sub‐networks with the highest scores (Figure 5C,D), and we suggest that these two modules might play a key role in the pathogenesis. Then, the cytohubba plug‐in was applied to obtain the top 20 scoring hub genes (Figure 5E). The miRNAs of TCGA ccRCC were screened, and we obtained a total of nine significant microRNAs (the number of samples with expression value of 0 was less than 10). The target genes of the nine miRNAs were identified using the TargetScan database, and correlation analysis was performed between the miRNAs and DEGs. Then, the genes with Pearson correlation coefficient greater than 0 were removed and taken with the target genes intersection. By removing genes with Pearson's *r* > 0, we sought to filter out relationships that do not align with the canonical inhibitory role of miRNAs in gene expression regulation. Finally, the miRNA‐mRNA regulatory network was obtained and visualized with Cytoscape (Figure 5B).

### 3.7 The relationship between immune cell infiltration and hub genes

**Figure 6:**
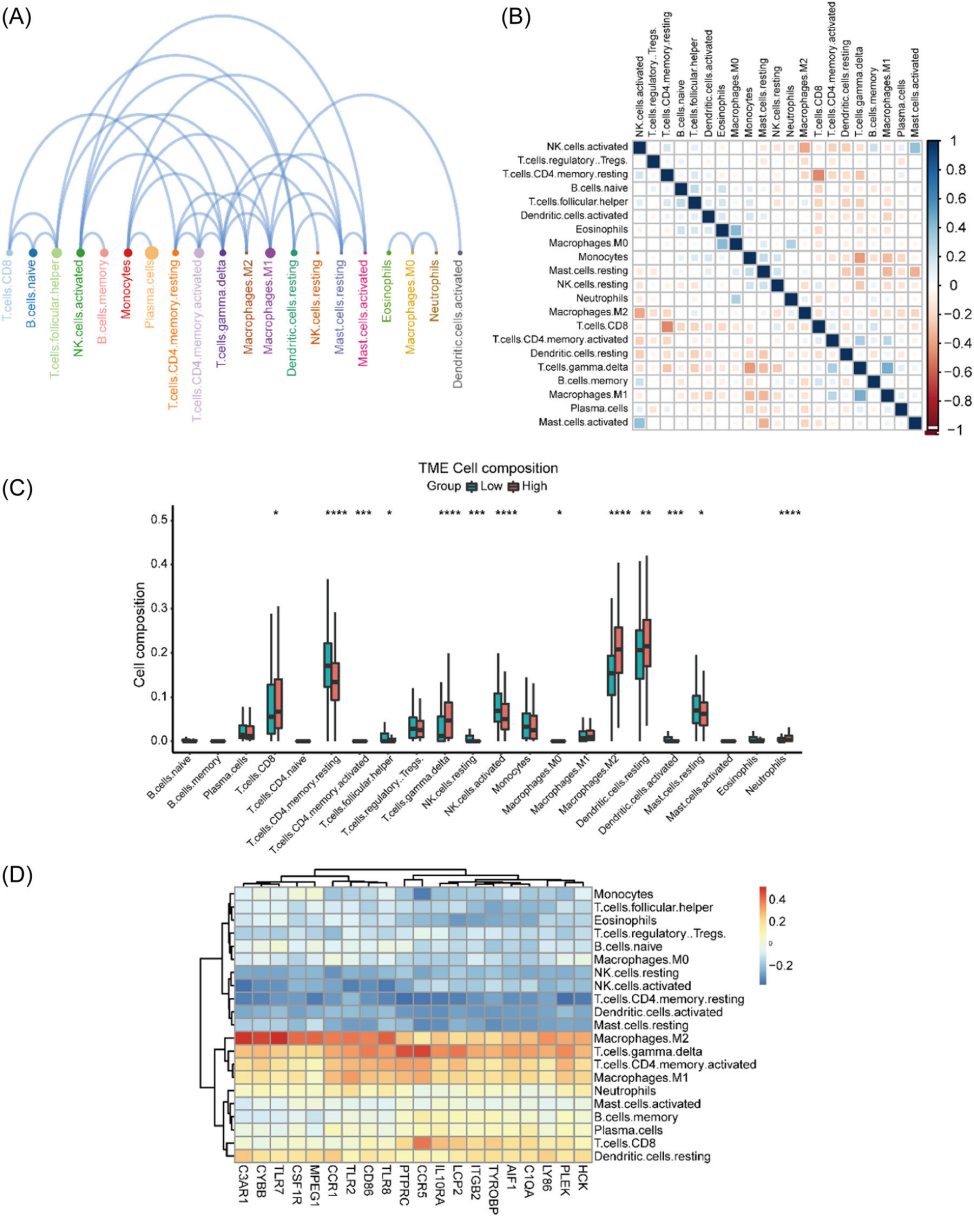
Visualization of immune cell infiltration and its correlation with diagnostic markers. (A) Interaction plot of 22 immune cell infiltrations; the size of the circle represents the strength of interaction with other immune cells, the larger the circle, the stronger the interaction with other immune cells; (B) correlation heatmap of 22 immune cell infiltrations; blue indicates positive correlation, red indicates negative correlation, the darker the color, the stronger the correlation; (C) Box plot of 22 immune cell infiltration ratio; red represents P2RY13 high‐expression group, blue represents P2RY13 low‐expression group; (D) correlation analysis of 22 diagnostic markers of immune cell infiltration; red represents positive correlation, blue represents negative correlation. *p < 0.05; **p < 0.01.

The results of 22 immune cell interactions (Figure 6A) showed that plasma cells had the strongest interactions with other immune cells (*p* < 0.05), while resting NK cells, resting mast cells, neutrophils, and M0 macrophages had weaker interactions with other immune cells. The 22 immune cell correlation heat map (Figure 6B) showed that M2 macrophages had a significant negative correlation with activated NK cells (*p* < 0.05); CD8 T cells had a significant negative correlation with resting memory CD4 T cells (*p* < 0.05); gamma delta T cells had a significant negative correlation with monocytes and NK cells (*p* < 0.05); gamma delta T cells had a negative correlation with monocytes, NK cells resting, and resting mast cells; activated mast cells had a significant positive correlation with activated NK cells (*p* < 0.05); and M1 macrophages had a significant positive correlation with gamma delta T cells (*p* < 0.05). The results of the box plot of immune cell infiltration differences (Figure 6C) showed that CD8 T cells, gamma delta T cells, M2 macrophages, resting dendritic cells, and neutrophil cells infiltrated more than the samples in the low expression group, while the infiltration of resting memory CD4 T cells, follicular helper T cells, resting NK cells, activated NK cells, activated dendritic cells, and resting mast cells was relatively low. Correlation analysis showed (Figure 6D) that immune cells were clustered into two groups: resting dendritic cells, M2 macrophages, memory B cells, plasma cells, gamma delta T cells, activated memory CD4 T cells, M1 macrophages, CD8 T cells, and activated mast cells were one group, as they all showed a significant positive correlation with 22 hub genes, while the remaining immune cells were grouped as they showed the opposite trend.

### 3.8 Identification of hub genes and survival analysis

**Figure 7:**
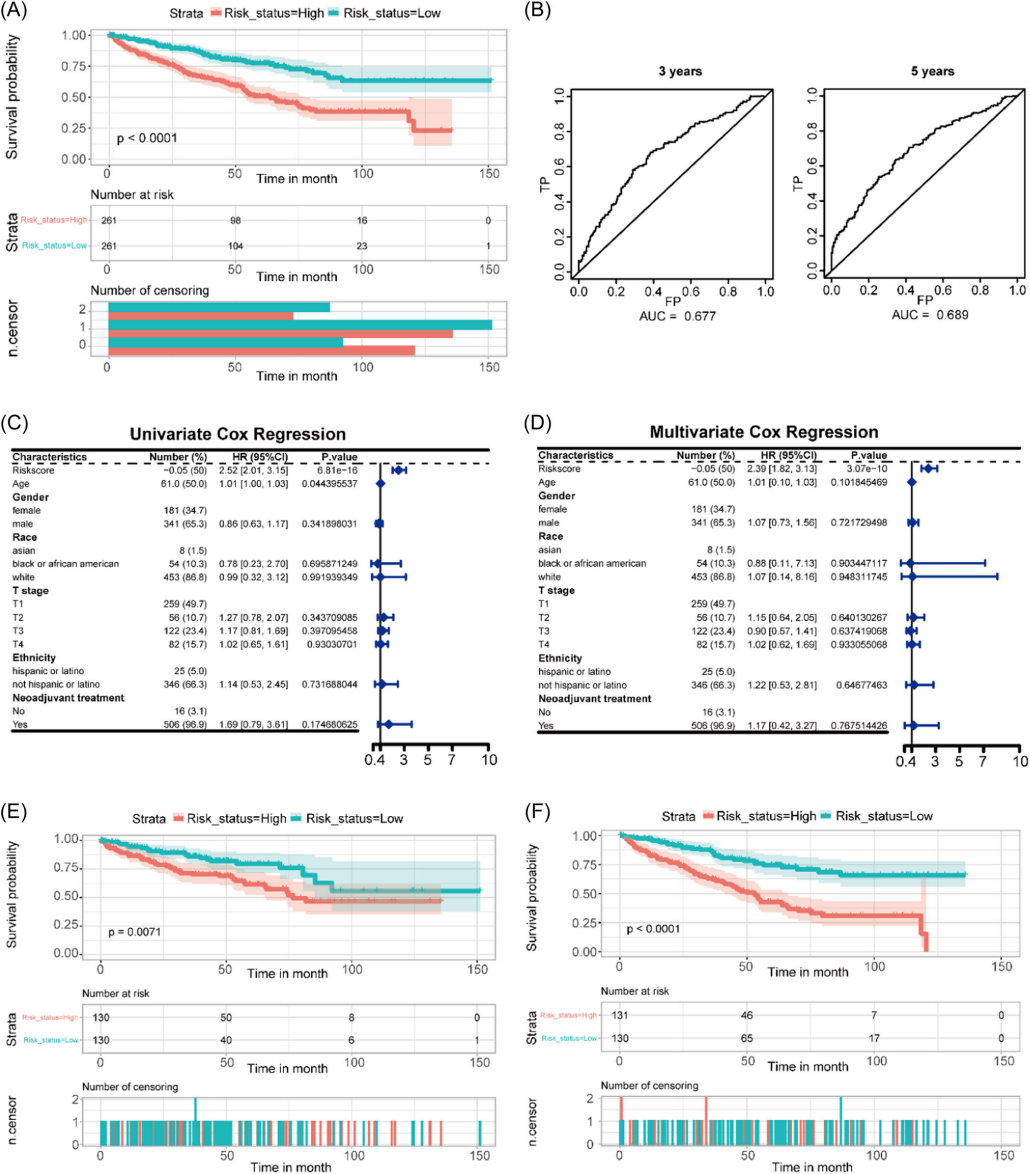
Prognostic gene screening and risk score calculation. (A) Survival analysis based on prognostic risk scores in TCGA ccRCC data set. (B, C) Time‐dependent ROC for prognostic risk scores; (D, E) Forest plot for Cox regression analysis; (F, G) survival analysis based on age grouping.

One‐way Cox proportional risk regression analysis was performed on the hub gene obtained from PPI network analysis, and genes with *p* < 0.05 were selected. A multi‐factor Cox proportional risk regression analysis was performed for the six obtained genes to construct a prognostic risk assessment model. The survival data used for our model was indeed derived from the TCGA database. According to the above model, the prognostic risk of each sample in TCGA was scored, and cutoff was the median value of the prognostic risk scores of patients in the training set. (Figure 7A). We then constructed a time‐dependent receiver operating characteristic curves (ROC) for prognostic risk score and patient survival and found that the AUC values were greater than 0.5 in TCGA ccRCC and GSE53757 (Figure 7B,C). Further, we subjected the prognostic risk scores to one‐way Cox proportional risk regression analysis and multi‐way Cox proportional risk regression analysis with the clinical characteristics of the patients, and the results of the regression analysis are presented as forest plots (Figure 7D,E). As can be seen from the figure, age, and prognostic risk score were associated with patient prognosis in the univariate Cox proportional hazards regression analysis, and only prognostic risk score was associated with patient prognosis in the multivariate Cox proportional hazards regression analysis. To test whether the prognostic risk score was independent of this clinical factor, we performed survival analysis based on the prognostic risk score by dividing patients into younger and older groups and found that the prognostic risk score had a significant effect on patient prognosis in both the younger and older groups (Figure 7F,G).

### 3.9 Validation of prognostic genes and prognostic risk scores and their relationship with immunity

**Figure 8:**
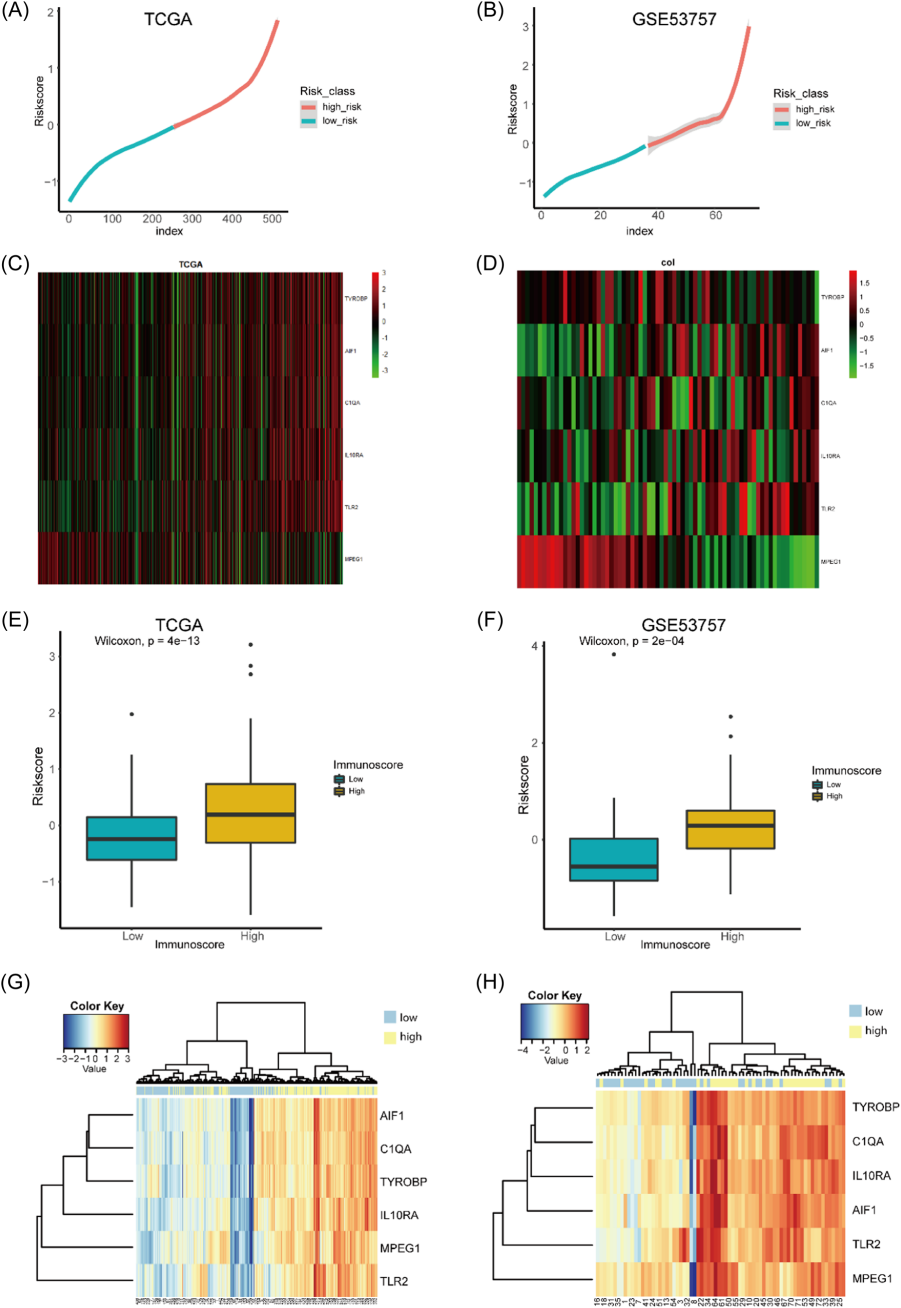
Prognostic genetic validation and immune analysis. (A, B) Risk score distributions in TCGA and GSE53757; (C) Heatmap after TCGA score ranking; (D) Heat map after GSE53757 score ranking; (E, F) Box plot of prognostic risk scores and immune scores in TCGA and GSE53757; (G) TCGA clustering heatmap; (H) GSE53757 clustering heatmap.

The patients were sorted from low to high risk based on the prognostic risk score, and as shown in Figure 8A–D, the expression of the marker of MPEG1 decreases as the score rises, whereas TYROBP, AIF1, C1QA, IL1ORA, and TLR2 expression tends to increase. Subsequently, to explore the diagnostic value of the six prognostic genes, we conducted hierarchical clustering analysis using the expression profiles of the six genes in TCGA ccRCC and GSE53757. As seen in Figure 8G,H, patients with high P2RY13 expression values were clustered into one category and those with low expression values into another. Subsequently, to explore the relationship between the prognostic risk score and immunity, we obtained the immune scores of patients using the R package ESTIMATE. The box plot (Figure 8E,F) shows that the high immune score group had a higher prognostic risk score, while a higher prognostic risk score corresponded to a worse prognosis, which is consistent with the findings of previous studies.^45^ This could be because in the high‐risk group, immune cells aggregate and attack other cells indiscriminately, leading to a worse prognosis.

### 3.10 IHC verified the expression of P2RY13

**Figure 9:**
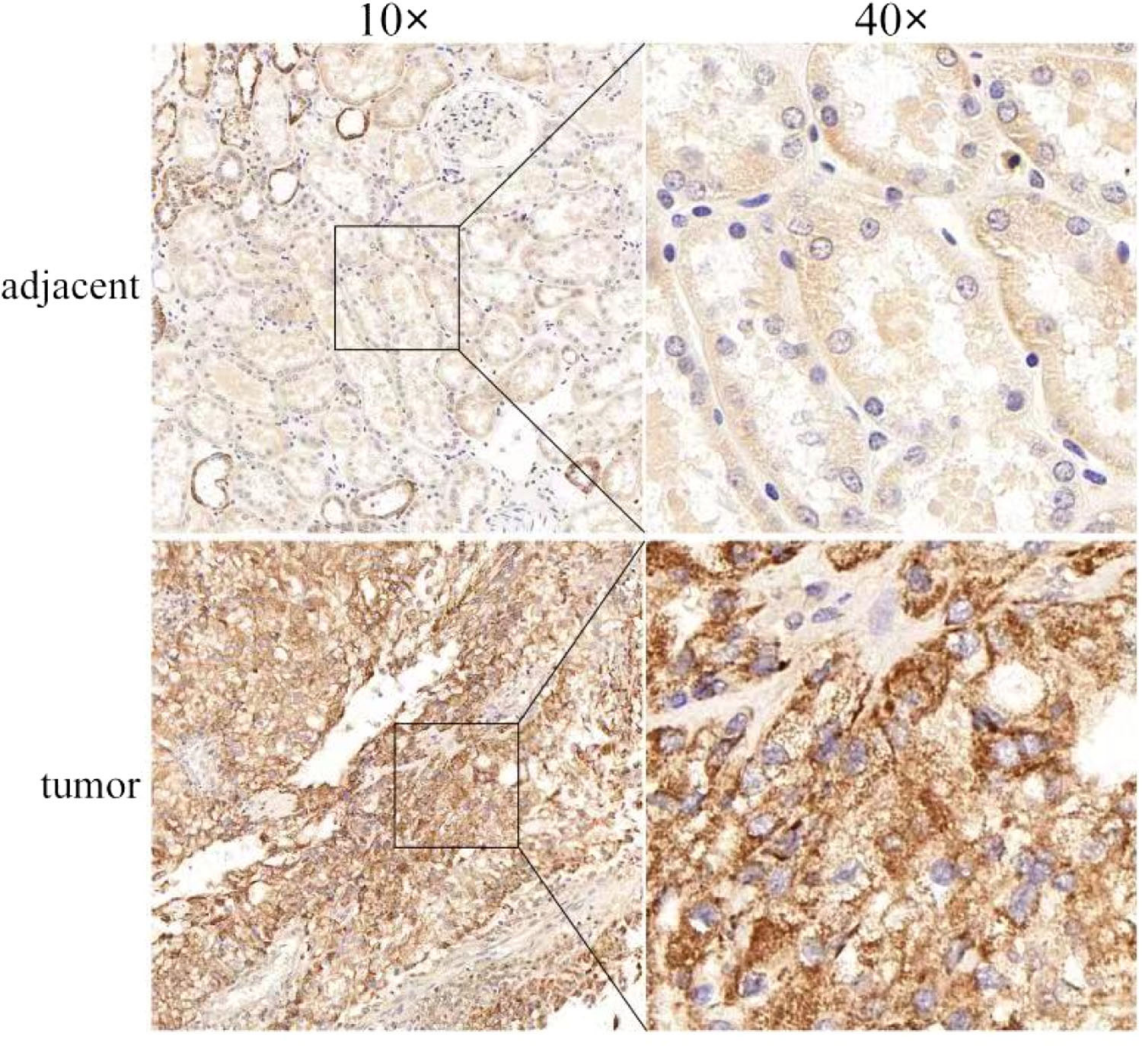
Immunohistochemistry verification of the expression of P2RY13 in ccRCC and paracancerous tissues. The results showed high expression of P2RY13 in ccRCC and low expression in paracancerous tissues under the microscope (×100 and ×400), *statistically significant (*p* < 0.05).

IHC showed that the expression of P2RY13 was significantly downregulated in paracancerous tissues, while P2RY13 was highly expressed in ccRCC, which was consistent with the results of our bioinformatics analysis (Figure 9). The detailed IHC results are shown in Supporting Information S1: Appendix 3.

## 4 DISCUSSION

ccRCC is a prevalent urinary tract cancer^11^ that accounts for up to 80% of primary renal tumors.^46^ ccRCC is a highly immuno‐infiltrative tumor in various clinical and genomic investigations,^47^ and the tumor immune microenvironment has become a research hotspot owing to its crucial role in ccRCC postoperative immunological monitoring.^48^ However, the tumor immune microenvironment remains poorly understood.^49^ Elucidating the molecular mechanisms underlying the immune microenvironment of ccRCC will help identify new therapeutic and chemopreventive targets for ccRCC; in this study, 345 DEGs were recovered from the GSE53757 gene expression matrix, while 1076 DEGs were extracted from TCGA gene expression matrix. Using the intersection of the difference sets of the two sets, 272 DEGs were identified. According to GO and KEGG, the DEGs were largely related to lymphocyte activation, inflammatory response, pattern recognition, and receptor function. The top 20 key genes in the PPI network linked with ccRCC were evaluated to identify key genes in the network. Resting dendritic cells, resting macrophages, M2 macrophages, memory B cells, plasma cells, T cells, M1 macrophages, and neutrophils were all related to the 20 hub genes substantially and favorably. In a study of the relationship between prognostic risk scores and immunity, higher immune scores were linked to higher prognostic risk scores.

P2RY13 is a member of the ADP receptor and G‐protein coupled receptor family and is theorized to be involved in hematopoietic and immunological processes.^50^ P2RY13 expression in steady‐state microglia was downregulated in a mouse model of neurodegenerative illness.^51^ A substantial negative connection was discovered in biopsies between acute inflammation scores and P2RY13 expression.^52^ P2RY13, a purinergic receptor, has been linked to a favorable prognosis in patients with lung cancer; however, the purinergic receptor P2RY13 is not linked to tumor recurrence^53^ In the sciatic nerve transcriptome, P2RY13 is enriched and well expressed.^54^ Furthermore, it has been shown that P2RY13 is a purinergic receptor gene that regulates microglia homeostasis and is involved in Alzheimer's disease susceptibility through inflammatory and neurotrophic mechanisms when activated by ADP in hepatocellular carcinoma cell lines.^55^, ^56^ However, no studies have been published on the function of P2RY13 in the development of ccRCC.

Of the top 20 genes obtained from the cytohubba plugin, the expression of C3ar1, a complement receptor signaling protein, was found to be increased in trNK cells.^57^ C3ar1 is active during complement activation, and C3ar1 mutant mice were shown to be protected from antigenic attack‐induced airway hyperreactivity.^58^ C3ar1 expression has been linked to renal tumors and normal tissues in some studies, while C3ar1 expression is elevated in renal carcinomas.^59^ In addition, although there is no direct evidence linking C3ar1 to obesity, indirect data from its ligand C3a suggest a relationship between C3ar1 and obesity‐related features,^58^ indicating that C3aR1 is a prospective obesity target.^60^ Evidence that microglia C3aR1 produces maladaptive alterations in response to neurogenic injury suggests that C3aR1 could be a potential therapeutic target for chronic pain management^61^ and that C3ar1 is implicated in the development of Th2 and asthma pathogenesis.^59^ CD86 expression in NK cells has previously been shown to confer an activating phenotype and increase cytotoxicity against tumors.^62^ CD86 overexpression was primarily observed in influenza NP cells, implying that CD86 upregulation is highly infection‐dependent.^63^ Tumor cells can express homologs of CD86 to prevent T‐cell activation and trigger death in colorectal tumors with low levels of CD86.^64^ In addition, low CD86 levels may play a role in immunological tolerance.^65^

Lymphocytic activation, inflammatory response, immune effector processes, pattern recognition receptor activity, immune receptor activity, membrane‐side and plasma membrane extrinsic, primary immunodeficiency, T‐cell receptor signaling pathway, and hematopoietic cell lineage pathways were all highly enriched in GO and KEGG. Activation of tumor lymphocytes is a key component of the household antitumor immune response.^66^ Studies have shown that tumors may induce tumor tissue apoptosis from activated lymphocytes and that tumor cells can escape and be removed from tumor tissue by acquiring lymphocytes.^67^ T cell tolerance is regulated by co‐inhibitory receptors on activated lymphocytes.^68^ The inflammatory response of tumors is a key^69^ factor. Chronic inflammation and inflammatory responses play a significant role at several stages of carcinogenesis, including tumor initiation, development, invasion, and metastasis^70^: Chronic inflammation increases tumor expansion and spreading, whereas acute inflammation suppresses tumor growth.^71^ Inflammation influences tumor radiation responses.^72^ The T‐cell receptor signaling pathway has been implicated in the molecular mechanisms of tumorigenesis^73^ and has been linked to immunological function.^74^ In addition, the T‐cell receptor signaling pathway has been linked to the development and progression of rheumatoid arthritis.^75^

GSEA was used to investigate the biological functions of the DEGs in ccRCC. Significant enrichment pathways included actin filament organization, immune response activation, adaptive immune responses, cytokine receptor interactions, adhesion, and neuroactive ligand‐receptor interactions. Because it modifies cytokine interactions to govern cancer growth and progression, cytokine–cytokine receptor interaction is a crucial immunological signaling mechanism^76^; inflammation, tumor immunology, and tumor metastasis are all influenced by cytokine–receptor interactions.^77^, ^78^ Immunosuppression has also been linked to cytokine–cytokine receptor interactions.^79^ The antitumor immunity of hosts consists of both innate and adaptive immune responses, but it is the adaptive immune system that is crucial for triggering powerful and highly targeted immune responses.^80^ Tumor cell death results in the release of tumor antigens, which trigger an adaptive immune response.^81^ Tumor cells can be killed by innate immune cells, which then trigger an adaptive immune response to eliminate the tumor.^82^ Tumor immunity, a key feature in tumor growth suppression, regulates tumor progression in most cancer types.^83^, ^84^

This study also showed that P2RY13 expression corresponds with immune infiltration in malignancies, particularly ccRCC. With respect to immune infiltration, box plots of differences in immune infiltration showed that CD8 T cells, gamma delta T cells, M2 macrophages, and dendritic cells had the strongest interactions with other immune cells, and while CD8 T cells and gamma delta T cells highly infiltrated, CD4 T cell, follicular helper T cell, NK cell, and dendritic cell infiltration had weak interactions. These immune cells correlated negatively with the 22 hub genes, while resting dendritic cells, M1 macrophages, neutrophils, CD8 T cells, and resting mast cells were positively correlated. Age and prognostic risk score were linked with patient prognosis in univariate Cox proportional risk regression; however, only prognostic risk score was associated with multivariate Cox proportional risk regression. The prognostic risk score had a substantial effect on patient prognosis in both the younger and older groups, independent of this clinical component. The predictive risk score showed that MPEG1 expression was reduced, whereas TYROBP, AIF1, C1QA, IL1ORA, and TLR2 expression increased. The diagnostic value of the six prognostic genes was then assessed, and their expression profiles were hierarchically clustered. Patients with high P2RY13 expression were grouped with those with low expression. High immune ratings were associated with a poor prognosis.

Although the current study increased our understanding of the association between P2RY13 expression and ccRCC, it still has certain limitations: First, many clinical aspects, such as details of the patients’ treatment, should be considered to completely understand the precise role of P2RY13 in the development of ccRCC. However, because the tests were carried out in different facilities, such data were either missing or were treated inconsistently in public databases. Second, the current work consists of a raw letter analysis with no validation studies. The functional mechanism between P2RY13 and ccRCC should be validated using wet‐lab qPCR, and cellular and animal experimental research. Third, this is a single histological study, and the functional understanding of P2RY13 is insufficient to clearly determine the direct mechanism of P2RY13 in renal clear cell carcinogenesis, as well as the next step of multi‐omics, particularly protein levels and functional mechanisms.

## 5 CONCLUSION

In summary, increased P2RY13 expression is related to tumor growth and poor survival and may promote tumorigenesis through abnormal inflammatory and immunological responses in ccRCC. More research is needed to show the biological effect of P2RY13 on ccRCC. Furthermore, more complete and consistent clinical data are required to understand the probable mechanisms of P2RY13 and its clinical applications in patients with ccRCC. This knowledge could aid in the discovery of new biomarkers that could lead to new insights into the clinicopathological relevance and molecular etiology of ccRCC.

## AUTHOR CONTRIBUTIONS

**Jie Chu**: Resources; software. **Wei Liu**: Validation. **Xinyue Hu**: Writing—original draft. **Huiling Zhang**: Data curation; formal analysis. **Jiudong Jiang**: Project administration; writing—review and editing. All authors have read and approved the final version of the manuscript. Huiling Zhang and Jiudong Jiang had full access to all of the data in this study and takes complete responsibility for the integrity of the data and the accuracy of the data analysis. We also declare that the supporting source/financial relationships had no such involvement.

## CONFLICT OF INTEREST STATEMENT

The authors declare no conflict of interest.

## TRANSPARENCY STATEMENT

The lead author Huiling Zhang affirms that this manuscript is an honest, accurate, and transparent account of the study being reported; that no important aspects of the study have been omitted; and that any discrepancies from the study as planned (and, if relevant, registered) have been explained.

## Supporting information

Supporting information.

Supporting information.

Supporting information.

Supporting information.

Supporting information.

Supporting information.

Supporting information.

## Data Availability

Publicly available data sets were analyzed in this study. This data can be found here: TCGA‐ccRCC, GSE53757.
