# Supplementary material for: P2RY13 is a prognostic biomarker and associated with immune infiltrates in renal clear cell carcinoma: A comprehensive bioinformatic study
Source: Health Sci Rep. 2023 Dec 1;6(12):e1646. doi: 10.1002/hsr2.1646 (PMC10691167; doi:10.1002/hsr2.1646)
Supplement: Supplementary file 1 — Supporting information. [file HSR2-6-e1646-s003.docx]

| Dataset | Patient | Control |
| --- | --- | --- |
| GSE53757 | 72 | 72 |
| TCGA ccRCC | 522 | 71 |
