# Supplementary material for: P2RY13 is a prognostic biomarker and associated with immune infiltrates in renal clear cell carcinoma: A comprehensive bioinformatic study
Source: Health Sci Rep. 2023 Dec 1;6(12):e1646. doi: 10.1002/hsr2.1646 (PMC10691167; doi:10.1002/hsr2.1646)
Supplement: Supplementary file 2 — Supporting information. [file HSR2-6-e1646-s007.docx]

| **Category** | **KEGG** | **Description** | **Count** | **LogP** |
| --- | --- | --- | --- | --- |
| KEGG Pathway | hsa05340 | Primary immunodeficiency | 13 | -16.85879782 |
| KEGG Pathway | hsa04660 | T cell receptor signaling pathway | 12 | -9.440302248 |
| KEGG Pathway | hsa04640 | Hematopoietic cell lineage | 17 | -16.1969871 |
| KEGG Pathway | hsa05152 | Tuberculosis | 21 | -15.54492396 |
| KEGG Pathway | hsa04658 | Th1 and Th2 cell differentiation | 16 | -14.76401691 |
| KEGG Pathway | hsa04514 | Cell adhesion molecules (CAMs) | 18 | -14.42583708 |
| KEGG Pathway | hsa04612 | Antigen processing and presentation | 14 | -13.66802295 |
| KEGG Pathway | hsa05150 | Staphylococcus aureus infection | 14 | -13.42503844 |
| KEGG Pathway | hsa05164 | Influenza A | 18 | -12.63300131 |
| KEGG Pathway | hsa05140 | Leishmania infection | 13 | -12.60877986 |
| KEGG Pathway | ko05140 | Leishmaniasis | 13 | -12.60877986 |
| KEGG Pathway | hsa04659 | Th17 cell differentiation | 15 | -12.56138426 |
| KEGG Pathway | hsa04145 | Phagosome | 16 | -11.03805989 |
| KEGG Pathway | hsa05321 | Inflammatory bowel disease (IBD) | 11 | -10.35232712 |
| KEGG Pathway | hsa05145 | Toxoplasmosis | 13 | -9.964166379 |
| KEGG Pathway | hsa05323 | Rheumatoid arthritis | 12 | -9.644056908 |
| KEGG Pathway | hsa05168 | Herpes simplex infection | 15 | -9.39178251 |
| KEGG Pathway | hsa05416 | Viral myocarditis | 9 | -8.277025706 |
| KEGG Pathway | hsa04672 | Intestinal immune network for IgA production | 8 | -7.659869185 |
| KEGG Pathway | hsa05330 | Allograft rejection | 7 | -7.149027759 |
