# Supplementary material for: P2RY13 is a prognostic biomarker and associated with immune infiltrates in renal clear cell carcinoma: A comprehensive bioinformatic study
Source: Health Sci Rep. 2023 Dec 1;6(12):e1646. doi: 10.1002/hsr2.1646 (PMC10691167; doi:10.1002/hsr2.1646)
Supplement: Supplementary file 3 — Supporting information. [file HSR2-6-e1646-s001.docx]

| **Category** | **Enrich Function** | **NES** | **enrichmentScore** | **pvalue** |
| --- | --- | --- | --- | --- |
| GO Biological Processes | GOBP_ACTIN_FILAMENT_ORGANIZATION | -1.371858578 | -0.501196439 | 0.000999001 |
| GO Biological Processes | GOBP_ACTIVATION_OF_IMMUNE_RESPONSE | -1.854080993 | -0.676842094 | 0.000999001 |
| GO Biological Processes | GOBP_ADAPTIVE_IMMUNE_RESPONSE | -2.060452367 | -0.753341272 | 0.000999001 |
| GO Biological Processes | GOBP_ADAPTIVE_IMMUNE_RESPONSE_BASED_ON_SOMATIC_RECOMBINATION_OF_IMMUNE_RECEPTORS_BUILT_FROM_IMMUNOGLOBULIN_SUPERFAMILY_DOMAINS | -1.92030456 | -0.708957352 | 0.000999001 |
| GO Biological Processes | GOBP_ADENYLATE_CYCLASE_MODULATING_G_PROTEIN_COUPLED_RECEPTOR_SIGNALING_PATHWAY | -1.443893912 | -0.533896187 | 0.000999001 |
| GO Biological Processes | GOBP_AMEBOIDAL_TYPE_CELL_MIGRATION | -1.386625609 | -0.507050159 | 0.000999001 |
| GO Biological Processes | GOBP_ANATOMICAL_STRUCTURE_HOMEOSTASIS | -1.325976119 | -0.484095237 | 0.000999001 |
| GO Biological Processes | GOBP_ANTIGEN_PROCESSING_AND_PRESENTATION | -1.814127751 | -0.670157442 | 0.000999001 |
| GO Biological Processes | GOBP_ANTIGEN_PROCESSING_AND_PRESENTATION_OF_PEPTIDE_ANTIGEN | -1.771707214 | -0.660052046 | 0.000999001 |
| GO Biological Processes | GOBP_ANTIGEN_RECEPTOR_MEDIATED_SIGNALING_PATHWAY | -1.869445806 | -0.690822275 | 0.000999001 |
| KEGG Pathway | KEGG_CYTOKINE_CYTOKINE_RECEPTOR_INTERACTION | -1.893236683 | -0.697736332 | 0.000999001 |
| KEGG Pathway | KEGG_FOCAL_ADHESION | -1.455459145 | -0.541873409 | 0.000999001 |
| KEGG Pathway | KEGG_NEUROACTIVE_LIGAND_RECEPTOR_INTERACTION | -1.362188741 | -0.501218263 | 0.000999001 |
| KEGG Pathway | KEGG_PATHWAYS_IN_CANCER | -1.373393918 | -0.505015879 | 0.000999001 |
| KEGG Pathway | KEGG_REGULATION_OF_ACTIN_CYTOSKELETON | -1.499964858 | -0.558573088 | 0.000999001 |
| KEGG Pathway | KEGG_CHEMOKINE_SIGNALING_PATHWAY | -1.885860353 | -0.70360203 | 0.001 |
| KEGG Pathway | KEGG_ENDOCYTOSIS | -1.426435362 | -0.533016113 | 0.001 |
| KEGG Pathway | KEGG_JAK_STAT_SIGNALING_PATHWAY | -1.736542803 | -0.652750126 | 0.001003009 |
| KEGG Pathway | KEGG_NATURAL_KILLER_CELL_MEDIATED_CYTOTOXICITY | -1.911938471 | -0.723907323 | 0.001005025 |
| KEGG Pathway | KEGG_SYSTEMIC_LUPUS_ERYTHEMATOSUS | -1.964620041 | -0.743316911 | 0.001005025 |
