# Supplementary material for: P2RY13 is a prognostic biomarker and associated with immune infiltrates in renal clear cell carcinoma: A comprehensive bioinformatic study
Source: Health Sci Rep. 2023 Dec 1;6(12):e1646. doi: 10.1002/hsr2.1646 (PMC10691167; doi:10.1002/hsr2.1646)
Supplement: Supplementary file 6 — Supporting information. [file HSR2-6-e1646-s004.docx]

Table 4. Differential expression of **P2RY13** in Renal cancer and adjacent tissues

|  | n | **P2RY13** expression | | Chi-square  Value | p value |
| --- | --- | --- | --- | --- | --- |
|  |  | High(%) | Low(%) |  |  |
| Renal cancer |  | 19 | 5 | 2.118 | 0.146 |
| Adjacent tissues |  | 15 | 19 |  |  |
|  |  |  |  |  |  |

* Statistically significant(p<0.05)


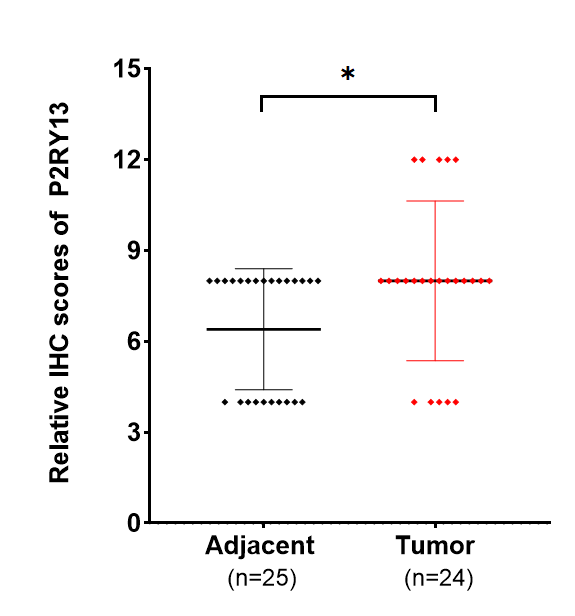


* Statistically significant(p<0.05)

Table5.Correlation between **P2RY13** expression and clinicopathological characteristics

|  | variables | P2RY13 expression | | total | p value |
| --- | --- | --- | --- | --- | --- |
|  |  | low | high |  |  |
| Age (year) |  |  |  |  | 1.000 |
|  | < 70 | 3 | 13 | 16 |  |
|  | ＞= 70 | 2 | 6 | 8 |  |
| Age (year) |  |  |  |  | 0.630 |
|  | < 65 | 3 | 8 | 11 |  |
|  | ＞= 65 | 2 | 11 | 13 |  |
| Sex |  |  |  |  | 1.000 |
|  | Female | 1 | 5 | 6 |  |
|  | male | 4 | 14 | 18 |  |
| Grade |  |  |  |  | 0.615 |
|  | II | 4 | 11 | 15 |  |
|  | III | 1 | 8 | 9 |  |
| T stage |  |  |  |  | 0.615 |
|  | I | 4 | 11 | 15 |  |
|  | II | 1 | 8 | 9 |  |
|  |  |  |  |  |  |

- * Statistically significant(p<0.05) , Since the total number of samples was less than 40, Fischer's exact test was used.
