# Supplementary material for: P2RY13 is a prognostic biomarker and associated with immune infiltrates in renal clear cell carcinoma: A comprehensive bioinformatic study
Source: Health Sci Rep. 2023 Dec 1;6(12):e1646. doi: 10.1002/hsr2.1646 (PMC10691167; doi:10.1002/hsr2.1646)
Supplement: Supplementary file 7 — Supporting information. [file HSR2-6-e1646-s002.docx]

**supplementary instruction**

**supplementary Table 1.** Related information of dataset platform (Affymetrix Human Genome U133 Plus 2.0 Array & Illumina)

**supplementary Table 2.** Top GO enrichment function obtained by Metascape

**supplementary Table 3.** Enrichment function of Top KEGG obtained by Metascape

**supplementary Table 4.** Top enrichment function obtained by GSEA

**Supplement 1** The detailed enrichment results of GO and KEGG analysis.

**Supplement 2** The detailed enrichment results of GSEA analysis.

**Supplement 3** IHC verification of the expression of P2RY13, differential expression of P2RY13 in Renal cancer with adjacent tissues and the correlation between P2RY13 expression and clinicopathological characteristics.
